# Food and Nutrient Intake and Nutritional Status of Finnish Vegans and Non-Vegetarians

**DOI:** 10.1371/journal.pone.0148235

**Published:** 2016-02-03

**Authors:** Anna-Liisa Elorinne, Georg Alfthan, Iris Erlund, Hanna Kivimäki, Annukka Paju, Irma Salminen, Ursula Turpeinen, Sari Voutilainen, Juha Laakso

**Affiliations:** 1 School of Applied Educational Science and Teacher Education, University of Eastern Finland, Savonlinna, Finland; 2 The Genomics and Biomarkers Unit, National Institute for Health and Welfare, Helsinki, Finland; 3 Institute of Public Health and Clinical Nutrition, University of Eastern Finland, Kuopio, Finland; 4 Department of Clinical Chemistry and Hematology, Helsinki University Central Hospital Laboratory, Helsinki University Central Hospital, Helsinki, Finland; 5 HUSLAB, Helsinki University Central Hospital, Helsinki, Finland; 6 Finnish Safety and Chemicals Agency, Helsinki, Finland; Max Delbrueck Center for Molecular Medicine, GERMANY

## Abstract

**Background:**

Vegetarian and vegan diets have become more popular among adolescents and young adults. However, few studies have investigated the nutritional status of vegans, who may be at risk of nutritional deficiencies.

**Objective:**

To compare dietary intake and nutritional status of Finnish long-term vegans and non-vegetarians.

**Methods:**

Dietary intake and supplement use were estimated using three-day dietary records. Nutritional status was assessed by measuring biomarkers in plasma, serum, and urine samples. Vegans’ (n = 22) data was compared with those of sex- and age-matched non-vegetarians (n = 19).

**Results:**

All vegans adhered strictly to their diet; however, individual variability was marked in food consumption and supplementation habits. Dietary intakes of key nutrients, vitamins B12 and D, were lower (P < 0.001) in vegans than in non-vegetarians. Nutritional biomarker measurements showed lower concentrations of serum 25-hydroxyvitamin D3 (25(OH)D3), iodine and selenium (corrected for multiple comparisons, P < 0.001), Vegans showed more favorable fatty acid profiles (P < 0.001) as well as much higher concentrations of polyphenols such as genistein and daidzein (P < 0.001). Eicosapentaenoic acid proportions in vegans were higher than expected. The median concentration of iodine in urine was below the recommended levels in both groups.

**Conclusions:**

Long-term consumption of a vegan diet was associated with some favorable laboratory measures but also with lowered concentrations of key nutrients compared to reference values. This study highlights the need for nutritional guidance to vegans.

## Introduction

Finnish adolescents and adults in western societies are increasingly adopting vegetarian diets [1, 2]. In Finland, the estimated proportion of adult vegetarians is 4.1% [3], and vegans are estimated to comprise ~1% of vegetarians [4]. People become vegetarians for various reasons; however, ideological reasons are more common than health-related ones [5].

Few studies have investigated the nutritional status or health of vegans and raw food consumers; however, those available have been valuable for planning and evaluating the nutritional status of vegans [6, 7, 8, 9].The recommendation for vegans is to maintain a variety of foods of vegetarian origin in their diet and to ensure adequate intakes of vitamin B12, vitamin D, riboflavin, calcium, iron, zinc, iodine, selenium, and protein by consuming either supplements or enriched foods. The consumption of seaweed is not recommended as a source of iodine as its iodine content is highly variable [10].

In general, the Finnish population has an appropriate nutritional status. However, it has been shown that lower levels (<50 nmol/l) of vitamin D still exist [11]. Therefore, recommendations regarding the fortification of fat spreads and milk products were changed during the last decade; furthermore, studies investigating the effect of the changed recommendations are now underway. Iodine is another nutrient whose intake may be compromised in the general population, mainly because the consumption of iodized table salt has decreased markedly. In 2015, a new recommendation was issued to return iodine intakes to the recommended levels [12].

It is now easier for vegans to compose an adequate vegan diet than it was a decade ago as the market has become more versatile in this respect. Furthermore, the number of fortified products has increased, dietary supplements and new vegetarian convenience foods are more readily available, and there is a greater awareness of what an appropriately planned vegetarian diet means [13]. However, previous studies have shown that owing to different food choices, vegetarians’ food and nutrient intakes vary among subgroups (e.g., semi-vegetarians, lactovegetarians, and vegans) [14]. In addition, even within the same subgroup, for example, vegans, individual food intakes may vary considerably [5]. The factors known to influence vegetarians’ food intake are, among others, the ethos of the vegetarian regimen; knowledge of a balanced, healthful, vegan diet; variety in the vegetarian food supply; use of enriched food items; and supplementation habits [15, 16].

According to the American Academy of Nutrition and Dietetics, a vegan diet can be healthful provided the individual follows a few precautions [13]. Recent studies have shown that vegans face nutritional problems with respect to vitamin B12 [17, 18], 25-hydorxyvitamin D [19, 20, 21], iodine [22, 23], selenium [24], and long-chain n-3 fatty acid status [25, 26].

The present study was undertaken to assess the nutritional status and supplementation habits of Finnish long-term vegans, whom we expected to adhere strictly to the diet and to possess good knowledge of a healthy vegan diet. In the present study, particular emphasis was placed on key nutrients such as vitamin B12, vitamin D, iron, iodine, and long-chain n-3 fatty acids because their intake may be insufficient in vegans. In addition, we measured nutritional biomarkers.

## Subjects and Methods

### 2.1. Subjects

We recruited the vegan participants (n = 22) through an advertisement published in the Finnish Vegan Association’s monthly newspaper and via an online discussion forum. Vegans were self-defined. The selection criteria were as follows: the participants had to (1) have followed a vegan diet for at least a year, [2] be between 18 and 50 years of age, [3] be apparently healthy, and [4] be nonusers of regular medications (except oral contraceptives or hormone replacement therapy). Non-vegetarian participants (n = 19) were recruited using the same media and criteria, except the dietary consumption. Further, we matched the non-vegetarian participants by age and sex. The study received the approval of the Ethical Committee of Kuopio University Hospital (69//2011). All participants provided their written consent to participate this study.

### 2.2. Dietary assessment

We assessed the consumption of foods at the baseline with a three-day food record using household measures. The food record sheets were blank and consisted of only an example menu. The participants received instructions on how to complete this record, and a dietician checked the completed food records. The participants also filled out a questionnaire concerning their long-term eating habits, including nutrient supplementation. The question concerning the consumption of a special diet included three vegetarian options (vegetarian, lacto-vegetarian and vegan) where vegetarian option was regarded as a semi-vegetarian option. The duration of the present diet was asked on a separate question.

We calculated the food and nutrient intakes using the Finnish Nutrica® software version 2.5, which is compiled mainly using Finnish values for the nutrient composition of foods. These calculations take into account the loss of vitamins during food preparation. This software was developed at the Research Centre of the Social Insurance Institution of Finland [27]. We obtained the nutrient compositions of the foods in the Nutrica® software from analyses carried out in the 1990s. The Nutrica® software contains the latest data on the vitamin contents of fruits and vegetables; furthermore, it provides a comprehensive database comprising more than 1,300 food items and dishes and 30 nutrients.

Moreover, we grouped the food items as follows: whole grain products (including rye products), rye products, rice, pasta, vegetables, root vegetables, potatoes, pulses, nuts, fruits, berries, fruit juices, berry juices, coffee, and tea. Specifically, the whole grain products included different breads, flakes, wheat bran, germ, and muesli products and excluded refined flour products. The rye products included different rye breads, rye flour, flakes, bran, and malt. The rice and pasta groups included both whole grain and refined products, which we did not include in the whole grain products. The vegetable group included all fresh and frozen vegetables and excluded pickled and canned vegetables. The root vegetables (the most commonly consumed root vegetables in Finland are carrots, Swedish turnip [swede], turnip, and beetroot) included all roots, except potatoes, which constitute a separate variable. The fruits included fresh, canned, and dried fruits and fruit nectars, while juices belonged to fruit juices. The berries included all fresh and frozen berries, crushed lingonberries, and lingonberry jam, which, in Finland, are usually prepared without sugar. The coffee and tea variables included all coffee and tea drinks.

### 2.3. Sample collection and biochemical analyses

We collected fasting peripheral venous blood samples and centrifuged them to separate the plasma and the serum, and we stored them at -70°C before the analysis. Furthermore, we collected urine samples 24 h prior to phlebotomy and divided them into aliquots. For one of the subjects, only a spot urine sample was available. We carried out the hematological and chemical analyses at Vitalab (hematological data), Helsinki University Central Hospital (folate, vitamin B12, vitamin D, and homocysteine), and National Institute for Health and Welfare (α-tocopherol, β-carotene, selenium, iodine, fatty acid profile, and polyphenols).

We used Siemens Advia 2120i and Advia 120 automatic cell counters to conduct the hematological analyses, which also involved the use of photometry to assess the participants’ Hb levels. The serum total cholesterol, HDL-cholesterol, and triglycerides were determined enzymatically using an Abbott Architect c8000 using commercial reagents from Abbott Laboratories (Abbott Park, IL, USA). LDL-cholesterol was calculated using the Friedewald formula.

To analyze the serum vitamin B12 and folate concentrations, we employed Abbot Architect i2000 and i2000SR analyzers (Abbott Diagnostics, Abbott Park, IL, USA) using two-step assays with automated sample pretreatment. Furthermore, we utilized Liquid Stable (LS) 2-part homocysteine reagent (Axis-Shield Diagnostics Ltd., Dundee, UK) with a Hitachi Modular P800 Chemistry analyzer (Hitachi Ltd., Tokyo, Japan) to analyze plasma homocysteine. We conducted all analyses according to the instructions of the manufacturers.

To analyze vitamin D, we used a previously unpublished method. 25-OH-D2 and 25-OH-D3 were obtained from Fluka; deuterium labelled internal standards (ISs) and 6,19,19-D3-25-OH-vitamin D3, from IsoSciences; and 26,27-D6-25-OH-vitamin D2, from Medical Isotopes Inc. We prepared stock solutions of 25-OH-D2, 25-OH-D3, and ISs in ethanol and diluted into methanol/water (70% v/v). We checked the standard solution concentrations by spectrophotometry (molar absorption coefficient: 18200 for both metabolites in ethanol at 265 nm). The imprecision for 25-OH-D2 and 25-OH-D3 was not higher than 7.0 and 7.1%CV, respectively.

To 150 μl of calibrator, serum, or quality control sample, we added 30 μl of IS working solution. After precipitating the proteins with 150 μl of methanol, we extracted the analytes with 3 mL of hexane. We dried the upper organic phase under nitrogen and dissolved the residue in 150 μl of 700 mL/L methanol/water. We injected 25 μl on an LC-MS/MS system comprising an API 4000 triple quadrupole mass spectrometer (AB Sciex) and an Agilent series 1200 HPLC system with a binary pump. To perform the separation, we employed a Discovery HS F5 column (2.1 × 100 mm, 3 μm; Waters, 30°C, flow rate of 300 μl/min). We employed a linear methanol/water gradient (0 min, 70% methanol; from 6.7 min to 10 min, 90% methanol; and from 10.2 min to 15 min, 70% methanol). The column was directly connected to the electrospray ionization probe.

We detected 25-OH-D_2_, 25-OH-D_3_, and ISs in the multiple reaction monitoring mode (25-OH-D_2_, m/z 413 to m/z 395; 25-OH-D_3_, m/z 401 to m/z 383; and ISs, m/z 405 to m/z 386 and m/z 419 to m/z 401). We obtained the data and processed it using the Analyst Software (Ver. 1.4, Sciex). All results were generated in the positive-ion mode (entrance potential: 10 V, declustering potential: 65 V, collision cell exit potential: 11 V, collision energy potential: 14 V). The front-end electrospray settings for the MS/MS ionization source were as follows: curtain gas, 25; GS1, 30; GS2, 30; CAD, 6; probe temperature, 350°C; and ion spray voltage, 5500 V. For all MS/MS experiments, we optimized all mass calibration and resolution adjustments on both the resolving quadrupoles using a polypropylene glycol solution with an infusion pump. We performed collisionally activated decomposition MS/MS with nitrogen as a collision gas.

We analyzed plasma α-tocopherol and β-carotene by reverse-phase HPLC, as previously described [28]. The imprecision between series for α-tocopherol was 3.7% and that for β-carotene, 5.2%. We analyzed plasma selenium using an electrothermal atomic absorption spectrometric method modified from [29] and [30]. We diluted a 50 μL aliquot of plasma fivefold with a solution containing 530 mg ascorbic acid and 0.2% Triton X-100. Then, we injected 15 μL of the diluted sample + 4 μL of 0.79 mmol/L Pd in 120 mmol HCl into a graphite tube. The instrument used was Perkin-Elmer 600 with Zeeman background correction. The imprecision between series was 4.4%, and the mean inaccuracy from analyzing serum standards (ClinCheck, Recipe, Munich,Germany) was 2.3%. We analyzed the urinary iodine concentrations using a microplate method [31]. The between series imprecision was 9%. Participation in the EQUIP (CDC, Atlanta) urinary iodine quality program showed a mean bias of -5.7%.

We utilized gas chromatography to determine serum fatty acids. We extracted the serum using dichloromethane-methanol (2:1, v:v) [32] and transesterified the fat yield with dry methanol (5% H_2_SO_4_) [33]. We analyzed the methylated fatty acid composition using an Agilent 6890 gas chromatograph (Hewlett Packard, Palo Alto, CA, USA) with a split injector and hydrogen as the carrier gas. We employed a DB-225 column (length: 30 m, I.D.: 0.32 mm, phase layer: 0.25 mm; Agilent J&W GC, Palo Alto, CA, USA). The temperature was varied from 160°C to 230°C. We normalized the percentage composition of fatty acid methyl esters to 100%. The between series variability of this method is 2%–7% for fatty acid peaks over 1% and 6%–18% for smaller peaks.

We determined the serum polyphenols, including genistein and daidzein, by gas chromatography and mass spectrometry after enzymatic hydrolysis using a modification of a previously described method [34]. The between series imprecision of polyphenols was 5%–9%.

### 2.4. Statistical methods

We expressed the data as mean ± SD (min-max); only the biochemical data was expressed as *medians* (with 25^th^ and 75^th^ percentiles). Differences between the groups were analyzed using the Mann-Whitney *U* test. Due to possible bias in statistical significances between the groups we made Bonferroni corrections for each group of parameters. Corrected threshold for statistical significance was calculated separately for food [30], nutrient [33], biochemical [30] and fatty acid [20] parameter group. We also made power calculations to find out how sensitive our data is to notice the possible difference between the groups in key nutrients such as vitamin B12, vitamin D, and iodine. We performed all statistical analyses using PASW Statistics 18 (2009, SPSS Inc.).

## Results

The sample comprised 22 vegans (6 men, 16 women) and 19 non-vegetarians (8 men, 11 women). There were no statistically significant differences between the groups in terms of age, body mass index (BMI), tobacco smoking, or frequency of alcohol consumption during the previous 30 days. On average, the vegan men had adhered to their diet for 7 years (range: 2–11) and the vegan women, for 9 years (range: 5–16) (Table 1). In addition, both the vegans and the non-vegetarians commonly used nutritional supplements (91% and 78%, respectively). The most frequently used supplements were multivitamin formulations, vitamin D, vitamin B12, and calcium supplements. Among the vegan subjects, 91% took vitamin B12 supplements and 77% took vitamin D supplements. Among the non-vegetarians, 78% took vitamin D supplements. Many of the non-vegetarian subjects used several supplements in highly variable amounts, as shown in Table 1.

**Table 1:** Basic characteristics of the study population.

	Vegans (n = 22)	Non-vegetarians (n = 19)	P-value for difference^1^
Age (y) ^2^	33 (24–50)	35 (24–52)	0.424
Gender			
Females	16	11	
Males	6	8	
BMI (kg/m^2^) ^2^	21.9 (18.1–25.7)	22.6 (18.4–27.1)	0.528
Duration of vegan diet (y) ^2^	8.6 (2–16)	-	-
Multivitamin-mineralSupplement users (n)	7	12^3^	
Vitamin D supplement users (n)	15	10^3^	
Vitamin B12 supplement users (n)	16	3^3^	
Calcium supplement users (n)	6	1^3^	
Fish oil/n-3 supplement users (n)	1	8^3^	
Other nutrient supplement users (n)	7	12^3^	

^1^ P-values are for difference between diets (Mann-Whitney).

^2^ Values are means (min-max).

^3^ n = 17.

### 3.1. Food and nutrient intakes

The vegans had higher daily intakes of legumes (p ≤ 0.001), tofu and soy flour (p<0.001), and margarine (p<0.001) than the non-vegetarians did. According to Table 2, vegans consumed no meat and fish, and little if any amount of dairy or other animal-derived products. There were no observable statistically significant differences in the consumption of any other foods between the groups after adjusting the data with the new threshold for statistical significance obtained using Bonferroni correction for multiple comparisons (p<0.0015), Table 2. The vegans’ daily mean consumption of protein sources was as follows: soy milk (70 ± 125 g), soy yogurt (101 ± 187 g), soy groats (68 ± 70 g), wheat protein seitan (19 ± 36 g), and falafel (81 ± 90 g). Most vegan subjects consumed enriched plant food items such as soy and oat drinks daily.

**Table 2:** Food intake of vegans and non-vegetarians (g/d)^1^.

Food group		Vegans (n = 22)	Non-vegetarians (n = 15)	P-value for difference^2^
Grain and vegetables				
	Ryeflour products	84 ± 72 (0–281)	50 ± 54 (0–165)	0.119
	Whole grain	139 ± 128 (0–619)	65 ± 58 (0–181)	0.020
	Rice	25 ± 48 (0–151)	23 ± 29 (0–76)	0.453
	Pasta	33 ± 41 (0–133)	25 ± 35 (0–103)	0.593
	Potato	84 ± 79 (0–229)	60 ± 37 (0–117)	0.593
	Roots	67 ± 58 (0–225)	103 ± 113 (0–457)	0.350
	Vegetables^3^	277 ± 186 (78–839)	246 ± 159 (87–660)	0.748
	Pulses and nuts	11 ± 20 (0–83)	5 ± 8 (0–24)	0.366
	Legumes	81 ± 90 (0–390)	15 ± 12 (0–41)	0.001*
	Tofu and soyflour	68 ± 70 (0–313)	2 ± 6 (0–23)	p<0.001*
	Soybeans	7 ± 18 (0–84)	0 (0–0)	0.036
	Mushrooms	12 ± 14 (0–53)	2 ± 5 (0–17)	0.010
Fruits and berries				
	Fruits	223 ± 187 (0–638)	266 ± 185 (0–668)	0.531
	Berries	31 ± 44 (0–138)	114 ± 164 (0–587)	0.112
	Fruit juices	103 ± 169 (0–700)	38 ± 111 (0–433)	0.304
	Berry juices	34 ± 73 (0–217)	4 (0–33)	0.531
Fats and oils				
	Butter	0.34 ± 1.11 (0–5)	6 ± 5 (0–16)	p<0.001*
	Margarine	28 ± 20 (5–71)	10 ± 5 (3–19)	p<0.001*
	Oils	9 ± 10 (0–33)	12 ± 10 (0–34)	0.202
	Other fats^4^	14 ± 18 (0–60)	4 ± 5 (0–15)	0.262
Milk products				
	Milk	59 ± 83 (0–326)	131 ± 130 (0–405)	0.049
	Sour milk, youghurt etc.	0.66 ± 3 (0–15)	69 ± 69 (0–227)	p<0.001*
	Cheese	7 ± 12 (0–40)	50 ± 35 (9–113)	p<0.001*
Meat and fish				
	Pork	0 (0–0)	28 ± 53 (0–169)	0.017
	Beef	0 (0–0)	100 ± 86 (0–353)	p<0.001*
	Sausages etc.	0 (0–0)	8 ± 15 (0–47)	0.181
	Fish, lower fat, (<0.5%)	0 (0–0)	18 ± 26 (0–75)	0.042
	Fish, higher fat	0 (0–0)	34 ± 35 (0–107)	p<0.001*
Drinks				
	Coffee	188 ± 203 (0–600)	324 ± 238 (0–667)	0.065
	Tea	431 ± 532 (0–2250)	307 ± 551 (0–2000)	0.152
Sugar and sweets				
	Sugar	15 ± 13 (0–42)	8 ± 9 (0–25)	0.070
	Sweets	11 ± 14 (0–43)	16 ± 20 (0–63)	0.453
	Chocolate	5.6 ± 10 (0–34)	4 ± 11 (0–41)	0.551

^1^ All values are mean ± SD (min-max).

^2^ P-values are for difference between vegans and non-vegetarians (Mann-Whitney).

^3^ Sum of fresh and frozen vegetables.

^4^ Include dressing and mayonnaise.

* Statistically significant after Bonferroni correction for multiple comparisons (the threshold of statistical significance is p<0.0015 when presented 33 parameters are taken into account).

There was no statistically significant difference in total energy intake between the vegans and the non-vegetarians; however, there were differences in nutrient intakes and essential nutrients. Specifically, the vegans obtained less, saturated fat, cholesterol, niacin, selenium, vitamin D, and vitamin B12 (p<0.001). Also the percent amounts of protein, carbohydrates, and SAFA intakes differed significantly between the groups (p<0.001), Table 3.

**Table 3:** Daily intake of nutrients of vegans and non-vegetarians^1^.

Dietary factor		Vegans (n = 22)	Non-vegetarians (n = 15)	P-value for difference^2^
Energy (MJ)		9.0 ± 2.6(4.2–13.4)	9.1 ± 2.7 (5.6–16.5)	0.867
Protein (g)		74 ± 30 (28–152)	103 ± 41 (61–230)	0.008
	(% of energy)	13.7 ± 2.8 (10–20.2)	19.1 ± 2.7 (14.4–23.8)	p<0.001*
Fat (g/day)		88 ± 37 (35–187)	109 ± 43 (60–209)	0.161
	(% of energy)	36.5 ± 7.2 (25.6–52.9)	44.9 ± 8.8 (27.5–64.3)	0.003
SAFA (g)		21 ± 9 (9–46)	39 ± 16 (21–74)	p<0.001*p<0.001*
	(% of energy)	8.6 ± 2.3 (4.6–13)	16.6 ± 4.7 (9.6–28.4)	p<0.001*
MUFA (g)		33 ± 18 (11–92)	38 ± 17 (20–77)	0.366
	(% of energy)	13.7 ± 4.8 (5.1–26)	15.6 ± 3.7 (9.1–22.6)	0.086
PUFA (g)		26 ± 11 (8–41)	19 ± 10 (9–44)	0.112
	(% of energy)	26 ± 11 (8–41)	7.8 ± 2.4 (4.3–13.6)	0.003
Cholesterol (mg)		44 ± 41 (0–142)	505 ± 439 (138–1822)	p<0.001*
Carbohydrates (g)		252 ± 67 (135–401)	182 ± 62 (83–287)	0.003
	(% of energy)	49.3 ± (32.7–60.3)	33.8 ± (14.2–53.7)	p<0.001*
Fiber (g/day)		41 ± 17 (17–84)	30 ± 15 (13–71)	0.039
Vitamin A (μg/RE)		1100 ± 756 (267–3675)	1744 ± 1402 (571–5953)	0.080
β-carotene (μg)		5807 ± 4367 (1213–21076)	7609 ± 8337 (1145–32802)	0.988
Vitamin D (μg)		5 ± 3 (1–15)	14 ± 8 (4–27)	p<0.001*
Vitamin E (mgL-TE)		20 ± 9 (7–36)	17 ± 10 (8–45)	0.237
Vitamin C (mg)		181 ± 134 (18–604)	236 ± 186 (43–757)	0.472
Thiamin (mg)		1.7 ± 0.9 (0.5–4.5)	1.5 ± 0.5 (0.9–2.4)	0.453
Riboflavin (mg)		1.5 ± 1.2 (0.5–6.6)	1.9 ± 0.9 (0.9–4.6)	0.028
Niacin (mg)		27 ± 11 (11–60)	41 ± 14 (25–81)	0.001*
Vitamin B12 (μg)		0.9 ± 0.8 (0–4)	8.7 ± 5.6 (3.4–24.7)	p<0.001*
Folate (μg)		586 ± 325 (203–1614)	402 ± 180 (177–871)	0.028
Calcium (mg)		1004 ± 623 (449–3451)	1117 ± 327 (651–1923)	0.056
Zinc (mg)		12 ± 4 (4–23)	16 ± 7 (8–35)	0.033
Iron (g)		21 ± 9 (8–46)	15 ± 7 (7–32)	0.026
Selenium (μg)		79 ± 65 (28–309)	149 ± 108 (57–404)	0.001*

^1^ All values are mean ± SD (min-max). Nutrient intake was calculated only from foods and drinks excluding dietary supplements.

^2^ P-values are for difference between vegans and non-vegetarians (Mann-Whitney).

*Statistically significant after Bonferroni correction for multiple comparisons (the threshold of statistical significance is p<0.0016 when presented 30 parameters are taken into account).

### 3.2. Nutritional status

The participants were healthy according to routine clinical chemistry measurements, except for three vegans who suffered from borderline anemia. The vegans also had lower ferritin stores, as shown in Table 4.

**Table 4:** Serum concentrations of nutrients, non-nutrients, and basic clinical data of vegans and non-vegetarians^1^.

Variable	Vegans (n = 21)	Non-vegetarians (n = 18)	P-value for difference^2^	Referencevalue of the laboratory
Vit B12 (pmol/L)	328 (238, 474)	508 (166, 661)	0.002	>140
Homocysteine (μmol/L)	8.6 (6.9, 10.8)	6.3 (5.3, 8.8)	0.069	<10.0
Folate (nmol/L)	21 (16, 31)	30 (19, 33)	0.257	5.3–40
Vitamin D (nmol/L) ^3^	54 (49, 69)	90 (75, 123)	p<0.001*	50–75
Vitamin D2	27 (19, 36)	2 (2, 3)	p<0.001*	
Vitamin D3	31 (15, 41)	90 (75, 105)	p<0.001*	
β-carotene (μmol /L)	0.75 (0.39, 1.39)	1.80 (1.09, 2.70)	0.001*	0.34–0.52
β-carotene: cholesterol (μmol/mmol)	0.18 (0.10, 0.33)	0.36 (0.20, 0.54)	0.005	
Vitamin E (μmol/L)	16.67 (14.8, 18.9)	21.1 (17.5, 28.1)	0.003	12–42
Vitamin E: cholesterol (μmol /mmol)	4.33 (4.14, 4.57)	4.66 (4.15, 5.18)	0.321	
Iodine (μg/L) ^4^	(4.6, 21.8)^5^	37.4 (17.7, 86.5)^6^	0.001*	100–200
Selenium (μmol/L)	(0.97, 1.37)	1.5 (1.33, 1.51)	0.001*	0.63–1.52
Hb (g/L)	139 (122, 144)	142 (135, 152)	0.174	117–155 F134–167 M
Hematocrit (%)	42 (39, 45)	44 (43, 47)	0.049	35–46 F39–50 M
Ferritin (μg/L)	26 (20, 39)	72 (16, 172)	0.011	5–100 F, 10–220M
Totalchol (mmol/L)	3.7 (3.4, 4.4)	4.6 (3.8, 5.4)	0.004	<5
HDL (mmol/L)	1.3 (1.0, 1.7)	1.6 (1.4, 2.1)	0.030	>1
LDL (mmol/L)	2.0 (1.8, 2.2)	2.6 (2.1, 3.5)	0.003	<3
Trigly (mmol/L)	0.75 (0.6, 1.1)	0.69 (0.53, 0.79)	0.165	<2
Leukocytes X10^9^/L	5.2 (4.5, 6.8)	4.9 (4.0, 5.4)	0.213	3.4–8.2
Erythrocytes X10^12^/L	4.4 (4.0, 4.8)	4.7 (4.9, 5.0)	0.032	3.9–5.2 F4.3–5.7 M
Trombocytes X10^9^/L	263 (221, 272)	273 (260, 344)	0.026	150–360
MCV (fL)	93 (90, 97)	93 (92, 96)	0.878	82–98
MCH (g/L)	31 (29, 32)	30 (29, 31)	0.184	27–33
MCHC (g/L)	329 (323, 334)	322 (318, 323)	0.028	320–355
Vanillic acid (nmol/L)	26.0 (14.9, 61.2)	18.5 (11.5, 26.2)	0.039	
Ferulic acid (nmol/L)	17.5 (11.6, 22.9)	9.8 (8.1 13.5)	0.031	
Caffeic acid (nmol/L)	18.1 (14.7, 30.8)	12.43 (11.4, 15.8)	0.012	
Genistein (μM)	0.360 (0.193, 1.576)	0.020 (0.020, 0.026)	p<0.001*	
Daidzein (μM)	0.306 (0.995, 0.912)	0.043 (0.026, 0.065)	p<0.001*	

^1^ All values are medians; 25^th^ to 75^th^ percentiles in parentheses.

^2^ P-values are for difference between vegans and controls (Mann-Whitney).

^3^ Contains serum 25-hydroxyvitamin D2 (25(OH) D2) and D3 (25(OH) D3).

^4^ Urinary iodine.

^5^ n = 20.

^6^ n = 17.

* Statistically significant after Bonferroni correction for multiple comparisons (the threshold of statistical significance is p<0.0016 when presented 30 parameters are taken into account).

The vegans exhibited lower serum concentrations of vitamin B12, vitamin D (25-hydroxyvitamin D2 and D3) total cholesterol and LDL cholesterol, as well as plasma concentrations of selenium, β-carotene, and α-tocopherol compared to the non-vegetarians, as shown in Table 4. However, when adjusted to the new threshold of statistical significance (p<0.0016) only serum concentrations of vitamin D (25-hydroxyvitamin D2 and D3), and plasma concentrations of β-carotene, and selenium, and urinary output of iodine were significantly different between the groups. The median urine iodine concentrations were under the reference concentration in both study groups. We observed marked inter-individual variation in the intakes of energy and protein and in the concentrations of nutritional biomarkers.

The serum fatty acid profiles were markedly different in the vegans as compared to the non-vegetarians, as shown in Table 5. The vegans had lower proportions of saturated fatty acids and higher proportions of (n-6) PUFA (P < 0.001). In particular, the groups were differentiated by the absence of conjugated linoleic acid (CLA) in the serum of the vegans. The proportions of (n-9) monoenes (p<0.01) and linolenic acid (18:3 n-3) were higher in the vegans’ serum. The proportions of EPA (p<0.001) and docosahexaenoic acid (DHA) (p<0.001) were clearly lower in the vegans’ serum than in that of the non-vegetarians; however, the groups did not differ with regard to docosapentaenoic acid (DPA) (22:5 n-3). The serum concentrations of the isoflavones genistein (p<0.001) and daidzein (p<0.001) were considerably higher in the vegans than in the non-vegetarians.

**Table 5:** Fatty acids (% of total fatty acids) of vegans and non-vegetarians^1^.

Variable	Vegans (n = 21)	Non-vegetarians (n = 17)	P-value for difference^2^
14:0	0.53 ± 0.25 (0.25–1.42)	0.62 ± 0.18 (0.32–0.93)	0.031
15:0	0.09 ± 0.19 (0.05–0.13)	0.20 ± 0.04 (0.15–0.30)	p<0.001*
16:0	18.6± 1.98 (15.9–23.2)	20.5 ± 1.25 (18.8–24.0)	p<0.001*
16:1n-9	0.38 ± 0.13 (0.18–0.68)	0.25 ± 0.07 (0.13–0.37)	p<0.001*
16:1n-7	1.08 ± 0.51 (0.54–2.22)	1.54 ± 0.48 (0.85–2.72)	0.002
17:0	0.10 ± 0.09 (0.00–0.25)	0.27 ± 0.04 (0.19–0.35)	p<0.001*
18:0	6.81 ± 0.82 (5.10–8.33)	7.97 ± 1.07 (6.07–10.63)	0.001*
18:1n-9	22.1 ± 3.02 (14.4–27.4)	20.2 ± 1.72 (16.5–22.5)	0.004
18:1n-7	1.56 ± 0.26 (0.83–2.00)	1.49 ± 0.24 (0.94–1.90)	0.281
18:2n-6 (LA)	36.77 ± 3.78 (30.47–44.07)	31.74 ± 1.21 (29.76–34.35)	p<0.001*
18:3n-6	0.40 ± 0.18 (0.18–0.92)	0.21 ± 0.09 (0.11–0.42)	p<0.001*
18:3n-3 (LNA)	1.28 ± 0.58 (0.43–2.92)	0.73 ± (0.35–2.14)	0.011
9c, 11t-18:2 (CLA)	0 ± 0 (0–0)	0.18 ± 0.04 (0.12–0.25)	p<0.001*
20:1	0.28 ± 0.07 (0.2–0.48)	0.20 ± 0.06 (0.11–0.33)	p<0.001*
20:3n-6	1.57 ± 0.31 (1.01–2.25)	1.26 ± 0.50 (0.63–2.55)	0.004
20:4n-6	6.27 ± 1.40 (3.74–8.20)	6.87 ± 1.14 (4.88–9.27)	0.281
20:5n-3 (EPA)	0.63 ± 0.28 (0.26–1.28)	2.33 ± 1.60 (0.53–5.60)	p<0.001*
22:4n-6	0.31 ± 0.05 (0.18–0.39)	0.35 ± 0.07 (0.19–0.42)	0.024
22:5n-3 (DPA)	0.54 ± 0.14 (0.34–0.79)	0.62 ± 0.18 (0.26–0.95)	0.095
22:6 n-3 (DHA)	0.85 ± 0.30 (0.47–1.43)	2.25 ± 0.80 (0.98–3.97)	p<0.001*

^1^ All values are Mean ± SD (min-max).

^2^ P-values are for difference between vegans and non-vegetarians (Mann-Whitney).

* Statistically significant after Bonferroni correction for multiple comparisons (the threshold of statistical significance is p<0.0016 when presented 20 parameters are taken into account).

## Discussion

Our study investigated the dietary intake and nutritional status of vegan and non-vegetarian subjects. The vegans were compliant and adhered strictly to their diet. Despite the use of dietary supplements, vitamin D and/or iodine status was compromised in most vegans.

### 4.1. Plasma lipids, antioxidants, and isoflavones

Some health-related and nutritional measures were more favorable in vegans than in non-vegetarians. Most importantly, the serum total cholesterol was 20% and LDL cholesterol was 25% lower in the vegan group than in the non-vegetarian group. Furthermore, vegans showed a more favorable fatty acid profile and higher serum concentrations of certain polyphenols compared with the non-vegetarians. These findings were likely the result of high consumption of rapeseed oil and margarines as well as soy and rye products.

The vegans consumed relatively small amounts of fruit, berries, nuts, and root vegetables, which was the likely cause of the lower serum concentrations of β-carotene (p = 0.001) and α-tocopherol (p = 0.003) compared to the non-vegetarians. However, after calculating the ratio of serum β-carotene and α-tocopherol to cholesterol concentration, and when adjusted to the new threshold of statistical significance obtained in Bonferroni calculation for multiple comparisons (p<0.0016) the differences in these antioxidant nutrients were not statistically significant. The poorer antioxidant vitamin status of vegans disagrees with the findings of earlier studies [35]. This is likely because the non-vegetarian subjects of this study were health conscious, as shown by their high consumption of different vitamin and mineral supplements as well as fruits and berries.

### 4.2. Vitamins B12 and D

Despite the use of nutritional supplements, the serum vitamin B12 concentrations in the vegans were lower compared to the non-vegetarians (p = 0.002); however, only 5% of vegans had serum vitamin B12 concentration below 140 pmol/L. It therefore appears that the consumption of vitamin B12 supplements, which 91% of the vegan subjects consumed, maintained their serum vitamin B12 concentrations within the reference limits. The onset of deficiency symptoms such as neuropsychiatric disorders and megaloblastic anemia usually occurs in 5–10 years when the serum vitamin B12 concentration is below 150 pmol/L [36].

The serum total concentration of vitamin D (25-hydroxyvitamin D2 and D3) was 34% lower in the vegans than in the non-vegetarians. However, the vegans had higher concentrations of 25-hydroxyvitamin D2 (p<0.001). The fraction of subjects having serum vitamin D concentration >75 nmol/L, which is the level proposed by some researchers to be optimal for preventing adverse health conditions [37], was 10% in vegans and 78% in non-vegetarians. In addition, more vegans had a serum vitamin D concentration ≤50 nmol/L as compared to the non-vegetarians (24% vs. 6%). The reasons for the marginal vitamin D status are presumably neglecting supplementation (23% of vegans), irregular supplementation, and, possibly, the time of sampling. 25-hydroxyvitamin D3 (calcidiol) concentrations are typically lowest during the winter [38]. Similar lower calcidiol concentrations were reported in Finnish, British, and Vietnamese vegans. [8, 9, 20, 21]

### 4.3. Iodine and selenium

All vegan subjects and 91% of the non-vegetarian subjects had iodine concentrations lower than the WHO’s limit for mild iodine deficiency (<100 μg/L urine). These data indicate that iodine intake may be insufficient in the Finnish population but particularly so in vegans, who do not consume milk products, the main source of iodine in many countries. Previously, goiter caused by iodine deficiency was common in Finland. However, after the fortification of table salt and cattle feed with iodine started some fifty years ago, iodine-deficiency-related goiter was eradicated. Today, the consumption of iodized table salt has decreased, partly because the food industry does not use iodized salt. Therefore, recommendations regarding iodine intake are not met by the general population [12]. Previous studies in vegans have also reported low urinary excretion of iodine. [6, 22, 23]

The serum selenium concentrations were lower in vegans than in non-vegans, however, on the whole, the values were similar to those found in countries that do not add selenium to fertilizers. It should be noted that Finland is the only country in the world that uses this strategy for supplementing the population with selenium [39]. The selenium intake was above the current nutrition recommendations [10] in both groups. The difference between groups is likely because dietary selenium is mainly obtained from animal products, which make up over 70% of the selenium intake in Finland [39].

### 4.4. n-3 fatty acids

Compared to the non-vegetarians, the proportions of C15:0, C17:0, and CLA, obtained mainly from milk products, were negligible in the vegans, indicating strict compliance with the vegan diet. The percentages of EPA and DHA of all fatty acids were respectively 0.6% and 0.9% in the vegans, and they were clearly lower than in the non-vegetarians. These differences were expected because vegans do not consume fish or fish oil products. However, the observed proportion of EPA in the vegans was still higher than expected. These results support the view that linolenic acid (LNA) is converted to EPA in humans. One may regard the vegans in this study as a high LNA population, as they consumed relatively high amounts of rapeseed oil, a common vegetable oil in the Nordic countries and a rich source of LNA. In countries consuming other types of vegetable oils, vegans would likely show even lower proportions of EPA and DHA in plasma. On the other hand, it should be noted that this issue is not straightforward, because linoleic acid (LA) and LNA compete for enzymes involved in fatty acid metabolism. A previous study among Kenyan Maasai [40] showed that despite a negligible intake of EPA and DHA, the proportion of DHA in red blood cells (RBCs) was no less than half that of a German sub-cohort. The authors speculated that a low intake of LA could also be advantageous and favor the endogenous conversion of LNA to DHA at a state of competition between n-3 and n-6 fatty acids.

### 4.5. Strengths and limitations of this study

Few studies have investigated the nutritional status of long-term vegans; therefore, our study gives important new information about these issues. However, this study has some limitations. Most importantly, the sample size was small and the results should therefore be confirmed in a bigger and more representative sample. Vegans were also self-defined, however, their compliance was confirmed by analyzing blood fatty acid profile, and no traces of foods of animal origin were noticed. This sample represented rather well educated young adults whose primary reasons for adopting a vegan diet were animal welfare and environmental concerns rather than beneficial health effects. The dietary intake was assessed by food records, which are relatively accurate to population mean dietary intakes. In addition, we studied various nutritional biomarkers. The nutrient intake was calculated only from foods and drinks excluding dietary supplements; however, this was compensated when relevant nutritional biomarkers were analyzed. In addition to dietary habits, many other lifestyle factors such as physical activity, alcohol consumption, and smoking may have their impact on nutrition, and hence confound the data. However, no differences in these parameters were noticed between the groups. From a statistical viewpoint, performing multiple tests may result biased significances. In order to eliminate the possible bias we made Bonferroni calculations and used more specific thresholds for statistical significances in each group of parameters. Taking multiple comparisons problem into account, the observed power between group differences were 0.47 for iodine, 0.43 for vitamin B12 and 0.91 for vitamin D.

## Conclusions

This study corroborates the view that nutritional guidance is important to vegans and that vegan diets should be regularly supplemented with key nutrients. More emphasis should be placed on vitamin D, and iodine to ensure sufficient intakes. The results also indicate a more favorable lipid and fatty acid profile in vegans. However, as fatty fish is not consumed by vegans, we recommend using vegetable oils rich in LNA (18:3 n-3), such as rapeseed oil, to maximize EPA formation.
