# Correction: Food and Nutrient Intake and Nutritional Status of Finnish Vegans and Non-Vegetarians

**DOI:** 10.1371/journal.pone.0151296

**Published:** 2016-03-03

**Authors:** The PLOS ONE Staff

In Table 4, there are errors in the “Vegans (n = 21)” column for rows “Iodine (μg/L) ^4^” and “Selenium (μmol/L).” The publisher apologizes for the errors. Please see the corrected Table 4 here.

**Table 4:** Serum concentrations of nutrients, non-nutrients, and basic clinical data of vegans and non-vegetarians^1^.

Variable	Vegans (n = 21)	Non-vegetarians (n = 18)	P-value for difference^2^	Reference value of the laboratory
Vit B12 (pmol/L)	328 (238, 474)	508 (166, 661)	0.002	>140
Homocysteine (μmol/L)	8.6 (6.9, 10.8)	6.3 (5.3, 8.8)	0.069	<10.0
Folate (nmol/L)	21 (16, 31)	30 (19, 33)	0.257	5.3–40
Vitamin D (nmol/L) ^3^	54 (49, 69)	90 (75, 123)	p<0.001*	50–75
Vitamin D2	27 (19, 36)	2 (2, 3)	p<0.001*	
Vitamin D3	31 (15, 41)	90 (75, 105)	p<0.001*	
β−carotene (μmol/L)	0.75 (0.39, .39)	1.80 (1.09, 2.70)	0.001*	0.34–0.52
β−carotene: cholesterol (μmol/mmol)	0.18 (0.10, .33)	0.36 (0.20, 0.54)	0.005	
Vitamin E (μmol/L)	16.67 (14.8, 18.9)	21.1 (17.5, 28.1)	0.003	12–42
Vitamin E: cholesterol (μmol /mmol)	4.33 (4.14, 4.57)	4.66 (4.15, 5.18)	0.321	
Iodine (μg/L) ^4^	15.0 (4.6, 21.8)^5^	37.4 (17.7, 86.5)^6^	0.001*	100–200
Selenium (µmol/L)	1.1 (0.97, 1.37)	1.5 (1.33, 1.51)	0.001*	0.63–1.52
Hb (g/L)	139 (122, 144)	142 (135, 152)	0.174	117–155 F134–167 M
Hematocrit (%)	42 (39, 45)	44 (43, 47)	0.049	35–46 F39–50 M
Ferritin (μg/L)	26 (20, 39)	72 (16, 172)	0.011	5–100 F, 10–220M
Totalchol (mmol/L)	3.7 (3.4, 4.4)	4.6 (3.8, 5.4)	0.004	<5
HDL (mmol/L)	1.3 (1.0, 1.7)	1.6 (1.4, 2.1)	0.03	>1
LDL (mmol/L)	2.0 (1.8, 2.2)	2.6 (2.1, 3.5)	0.003	<3
Trigly (mmol/L)	0.75 (0.6, 1.1)	0.69 (0.53, 0.79)	0.165	<2
Leukocytes X10^9^/L	5.2 (4.5, 6.8)	4.9 (4.0, 5.4)	0.213	3.4–8.2
Erythrocytes X10^12^/L	4.4 (4.0, 4.8)	4.7 (4.9, 5.0)	0.032	3.9–5.2 F4.3–5.7 M
Trombocytes X10^9^/L	263 (221, 272)	273 (260, 344)	0.026	150–360
MCV (fL)	93 (90, 97)	93 (92, 96)	0.878	82–98
MCH (g/L)	31 (29, 32)	30 (29, 31)	0.184	27–33
MCHC (g/L)	329 (323, 334)	322 (318, 323)	0.028	320–355
Vanillic acid (nmol/L)	26.0 (14.9, 61.2)	18.5 (11.5, 26.2)	0.039	
Ferulic acid (nmol/L)	17.5 (11.6, 22.9)	9.8 (8.1 13.5)	0.031	
Caffeic acid (nmol/L)	18.1 (14.7, 30.8)	12.43 (11.4, 15.8)	0.012	
Genistein (μM)	0.360 (0.193, .576)	0.020 (0.020, .026)	p<0.001*	
Daidzein (μM)	0.306 (0.995, .912)	0.043 (0.026, .065)	p<0.001*	

1 All values are medians; 25th to 75th percentiles in parentheses.

2 P-values are for difference between vegans and controls (Mann-Whitney).

3 Contains serum 25-hydroxyvitamin D2 (25(OH) D2) and D3 (25(OH) D3).

4 Urinary iodine.

5 n = 20.

6 n = 17.

* Statistically significant after Bonferroni correction for multiple comparisons (the threshold of statistical significance is p<0.0016 when presented 30 parameters are taken into account).
